# Decompressive craniectomy for severe traumatic brain injury: clinical study, literature review and meta-analysis

**DOI:** 10.1186/s40064-016-3251-9

**Published:** 2016-09-20

**Authors:** Gene A. Grindlinger, David H. Skavdahl, Robert D. Ecker, Matthew R. Sanborn

**Affiliations:** 1 Maine Medical Center, 887 Congress Street, Suite 210, Portland, ME 04102 USA; 2 Surgical Residency Program, Maine Medical Center, Portland, ME USA; 3 Tufts University School of Medicine, Boston, MA USA; 4 Department of Neurosurgery, Maine Medical Center, Portland, ME USA

**Keywords:** Decompressive craniectomy, Severe TBI, TBI, DECRAN, Cerebral Edema

## Abstract

**Objective:**

To examine the clinical and neurological outcome of patients who sustained a severe non-penetrating traumatic brain injury (TBI) and underwent unilateral decompressive craniectomy (DC) for refractory intracranial hypertension.

**Design:**

Single center, retrospective, observational.

**Setting:**

Level I Trauma Center in Portland, Maine.

**Patients:**

31 patients aged 16–72 of either sex who sustained a severe, non-penetrating TBI and underwent a unilateral DC for evacuation of parenchymal or extra-axial hematoma or for failure of medical therapy to control intracranial pressure (ICP).

**Interventions:**

Review of the electronic medical record of patients undergoing DC for severe TBI and assessment of extended Glasgow Outcome Score (e-GOS) at 6-months following DC.

**Measurements and main results:**

The mean age was 39.3y ± 14.5. The initial GCS was 5.8 ± 3.2, and the ISS was 29.7 ± 6.3. Twenty-two patients underwent DC within the first 24 h, two within the next 24 h and seven between the 3rd and 7th day post injury. The pre-DC ICP was 30.7 ± 10.3 and the ICP was 12.1 ± 6.2 post-DC. Cranioplasty was performed in all surviving patients 1–4 months post-DC. Of the 29 survivors following DC, the e-GOS was 8 in seven patients, and 7 in ten patients. The e-GOS was 5–6 in 6 others. Of the 6 survivors with poor outcomes (e-GOS = 2–4), five were the initial patients in the series.

**Conclusions:**

In patients with intractable cerebral hypertension following TBI, unilateral DC in concert with practice guideline directed brain resuscitation is associated with good functional outcome and acceptable-mortality.

## Background

Traumatic brain injury (TBI) is a frequent cause of death from trauma with more than 50,000 deaths annually in the United States. 5.3 million survivors of TBI require some assistance in the performance of their activities of daily living. Death and severe disability following TBI is often attributable to the intracranial hypertension that is the result of brain swelling (Nirula et al. 2014; Gouello et al. 2014). Prevention of the secondary brain injury consequent to uncontrolled brain edema is the focus of medical management. Medical therapies aimed at lowering intracranial pressure (ICP) include hyperosmolar therapy, barbiturate coma, sedation, therapeutic hypothermia and ventricular drainage. The objective is the lowering of ICP to facilitate cerebral perfusion and oxygenation (Brain Trauma Foundation 2007). When the ICP becomes refractory to medical management (malignant cerebral edema) irreversible secondary brain injury likely has occurred and the mortality is nearly 100 % (Miller et al. 1977).

Hemicraniectomy was described as a treatment option for cerebral edema more than a century ago (Kocher 1901). It was first introduced in 1971 as a management option for traumatic subdural hematoma (Ransohoff et al. 1971). Early reports of decompressive craniectomy (DC) were disappointing as the procedure was used as a rescue attempt for malignant cerebral edema when medical therapy failed. Not surprisingly, the outcome of these patients was predictably poor with high mortality and unfavorable functional outcome in survivors (Ransohoff et al. 1971; Kjellberg and Prieto 1971; Venes and Collins 1975). Recently, there has been a renaissance of interest in DC, encouraged, in part, by its successful application to younger patients with large middle cerebral artery stroke (Juttler et al. 2007, 2014). In this population there is good evidence that early decompressive craniectomy improves both functional and mortality outcomes (Juttler et al. 2007). Furthermore, its application for military (penetrating/blast) injuries has resulted in some dramatic rescues (Ecker et al. 2011). Still, recent randomized control studies of bi-frontal craniectomy in blunt trauma showed decreased intensive care unit times and better control of ICP, but worse long-term outcome (Cooper et al. 2011). Even in the post hoc analysis when adjustments were made for pupillary reactivity, there were no benefits found with DC over medical management alone. Additionally, the 2009 Cochrane Review could not recommend DC in adult TBI patients (Sahuquillo 2006). Some recent series have had more encouraging results. Both survival and long term outcome were found superior to medical management especially when the procedure was performed within 48 h of injury rather than as a salvage procedure (Gouello et al. 2014; Aarabi et al. 2006; Eghwrudjakpor and Allison 2010; Whitfield et al. 2001).

In this observational study we report the therapeutic effects and encouraging long-term outcome results and decrease in mortality following unilateral DC in patients with severe TBI. A literature review and meta-analysis are included.

## Methods

This is a single-center, retrospective observational study of patient aged 16–70 who were managed by unilateral DC during the time period January 2010 to September 2015. The MMC Institutional Review Board (IRB) for Human Subjects approved this study (#4641x on 8/4/2015). Because this was a retrospective review analysis, the IRB waived the informed consent requirement. Patients who sustained a severe non-penetrating brain injury defined as a Glasgow Coma Score (GCS) of less than 8 were evaluated by the on-call Neurosurgeon either in the Emergency Department (ED) or soon after arrival in the ICU. All underwent CT brain imaging upon arrival to the Maine Medical Center (MMC) Trauma unit, a Level 1 Trauma Center in Portland, Maine and were either brought directly to the operating room (OR) for craniotomy or were admitted to the Neuro-Critical Care (NCC) Intensive Care Unit (ICU) for placement of an ICP monitor or external ventricular drainage (EVD) catheter. Patients brought directly to the OR from the ED underwent DC as a primary intervention when the bone flap could not be replaced due to hemispheric edema. Those patients not requiring immediate decompression were admitted to the NCC ICU. The NCC ICU has the capability of advanced neuro-monitoring including intracranial pressure monitoring (ICP), Integra Neuro-Sciences™, San Diego, CA, external ventricular drainage, cerebral brain tissue oxygen monitoring (PbtO2) (Raumedic^®^), cerebral blood flow (Hemedex^®^), temperature control (Arctic Sun 5000^®^) and continuous electroencephalography (cEEG). All patients admitted initially to the ICU whose GCS could not be explained by drug or alcohol intoxication, sedating medication or paralytic administration and had a survivable injury (patients with bilaterally dilated pupils were excluded) and were not made comfort measures only (CMO) by a surrogate health-care proxy underwent placement of an ICP monitor and stabilization and resuscitation according to the latest Brain Trauma Foundation Guidelines (Brain Trauma Foundation 2007). The intention was to keep and maintain the ICP at less than 20 mm Hg and cerebral perfusion pressure (CPP) at above 70 mmHg. To accomplish this, the clinical algorithm included head-of-bed elevation to 20–30 degrees, and maintenance of the neck in neutral position. The patient was kept normothermic by use of the Arctic Sun 5000^®^ if required. Fluid infusion was adjusted to avoid over-hydration, and hyponatremia was avoided or vigorously treated. Patients were kept well sedated. When ICP exceeded 20 mmHgl, mannitol 0.5–1.0 grams per kilogram or hypertonic saline 23.5 % (HTS) in aliquots of 50 cc was administered. If required, a 3 % HTS drip was started. Serum osmolarity (mosm) and serum sodium were strictly monitored and kept below a threshold of 320 mosm per kilogram, and 160 mEq sodium per liter. A ventriculostomy was placed if there was a target. Hyperventilation generally was not employed unless there were clinical signs of herniation. Elevated carbon dioxide levels were avoided by ventilator adjustment. If these measured failed to keep ICP below the threshold of 25 mm Hg, barbiturate coma was induced in some patients prior to craniectomy. The selection of patients for barbiturate administration was not based on age. When ICP exceeded 25 mmHg for greater than 30 min, strong consideration was given to proceed with DC. Patients were managed by a multidisciplinary team that included Surgical/Trauma Intensivists, Neuro-intensivists, Neurosurgeons, and NCC trained nurses. None had bilaterally dilated pupils. Initial CT scans and subsequent imaging was reviewed. Patients’ selected for DC underwent unilateral fronto temporoparietal (FTP) craniectomy following head injury either immediately, due to the nature of the traumatic injury or delayed due to failure of medical control of intracranial pressure. The technique of unilateral DC has been previously described (Huang and Wen 2010). Importantly, the craniectomy was extended into the temporal lobe base to release compression on the basilar cisterns (Huang and Wen 2010). Duroplasty was performed in all DC patients to allow for expansion of the edematous brain and to prevent adhesion formation of the subjacent brain (Gouello et al. 2014; Huang and Wen 2010; Yang et al. 2003; Mitchell et al. 2004). The bone-flap was stored at −70 °C for one to four months.

Data was collated from the electronic medical record (EMR), Surgical Critical Care database, neurosurgical office notes, neuro-rehabilitation center reports and the Radiology Imaging Archive system (IMPAX 6™). Patients were selected on the basis of specific coding for DC in the database. Demographic data included patient’s age, sex, and mechanism of injury, associated injuries, date of injury and comorbidities and date of DC. Clinical data included initial GCS, initial systolic blood pressure, CT imaging findings, modified Marshall CT grade, pre-craniectomy hypoxia or hypotension, Injury Severity Score (ISS), ICP (when present) prior to DC, and ICP following DC. Days ventilated, occurrence of infection or organ failure, ICU and hospital length of stay (LOS), and development of hydrocephalus, CSF leak, seizures or diabetes insipidus were recorded. All initial imaging was reviewed for injury description, presence of herniation, measurement of midline-shift, and cerebral dominance. Maximum antero-posterior diameter of the bone flap was measured on the initial post-operative CT scan. Long-term follow-up at 6 months was assessed using the extended Glasgow Outcome Score (e-GOS). Presence of late seizures, hemiplegia, late subdural hematomas, hydrocephalus, aphasia, or other complications related to DC was noted. Cranioplasty usually was performed 2–12 weeks post DC. Complications of cranioplasty including infection were recorded.

Demographics are presented as mean ± SD for continuous variables and number (n) for categorical variables. A unilateral logistic regression analysis and Pearson product-moment correlation were conducted to evaluate the association between clinical variables and a good Glasgow outcome score. Those variable found to be significantly associated with a good outcome (e-GOS of 6–8) were then analyzed by multiple logistic regression analysis. Finally, a meta-analysis of recent DC studies reported during the past decade is included to explore which clinical variables determine a good clinical outcome.

## Results

**Fig. 1:**
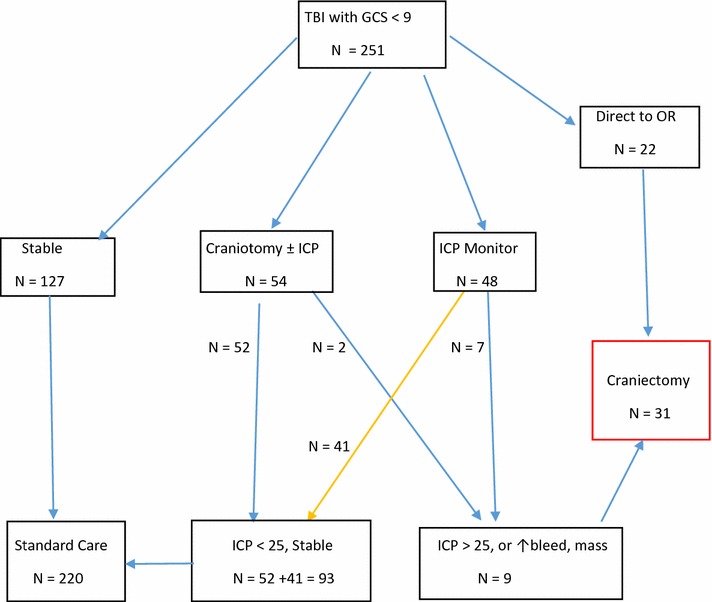
Algorithm for patient management

During the time period of this study, there were 752 patients admitted to the Surgical/NCC ICU with a TBI as an isolated injury or as a component of other non-CNS injuries (multi-trauma). 251 of these TBI patients presented or subsequently developed a GCS of <9, but in 127 the low GCS could be attributed to alcohol use, other drugs, sedation or administered paralytics. Of the remaining 124 patients, 31 underwent unilateral DC. The mean age of the DC patients was 39.3 ± 14.5 years (range 16–72 years). 26 patients were male. The initial GCS was 5.8 ± 3.2. Systolic BP was 133.8 ± 26.0 mmHg. The ISS was 29.7 ± 6.3 (Table 1). The mechanisms of injury of DC patients are tabulated in Table 2. Most sustained their injuries in falls or in motor vehicle crashes (MVC). Fourteen patients also sustained non-CNS injuries (multi-trauma). Twenty-eight of the 31 patients had midline shift on their initial head-CT scan (mean 8.7 ± 5.4 mm). Marshall scores varied from 2c to 6d on the pre-craniectomy imaging (Table 3). Three patients presented with a unilateral dilated pupil and 16 others with radiological signs of herniation. Patients on average spent 14.4 ± 6.5 days on ventilator support. The ICU length of stay (LOS) was 18.0 ± 7.4 days and the hospital LOS 51.3 ± 36.6 days. 22 patients underwent DC on the day of injury due to the magnitude of brain injury, significant midline shift, and/or herniation of the brain through a craniotomy done for evacuation of hematoma precluding replacement of the bone flap. Two patients underwent DC within 48 h of injury and seven between the 3rd and 12th day. Hence 22 patients underwent DC as the initial intervention and 9 others due to failure of medical control of cerebral hypertension, or for clinical or neuroimaging progression (Fig. 1). ICP was 30.7 ± 10.3 mmHg prior to DC and decreased to 12.1 ± 62 mmHg post-operatively. Barbiturates were required for ICP control prior to DC in 9 patients. The A-P diameter of the craniectomy bone flap was 15.5 ± 2.9 cm (Table 4). Of the remaining 210 patients, 54 underwent craniotomy for evacuation of extra-parenchymal or parenchymal hematomas. 48 others underwent placement of an ICP and brain oxygen monitor or ventriculostomy.

**Table 1:** Patient characteristics

Variable	Value
Age (y)^a^	39.3 ± 14.5
Male^b^	(26)
GCS (initial)	5.8 ± 3.2
SBP (mmHg)	133.8 ± 26.0
ISS	29.7 ± 6.5
Barbiturates use	(9)
Ventilator days (d)	14.4 ± 6.5
DC 1st 24 h	(21)
DC 24–48 h	(3)
DC 3–7 d	(7)
ICP pre-DC (mmHg)	30.7 ± 10.3
ICP post-DC (mmHg)	12.1 ± 6.2
Unilateral dilated pupil	(3)
Midline shift (mm)	8.7 ± 5.4
Herniation	(18)
ARDS	(7)
ARF	(1)
Cranioplasty	(29)
Dominant hemisphere	(14)

^a^Data presented as mean ± SD

^b^Data presented as number (n)

**Table 2:** Mechanism of injury

Mechanism	n
Fall from height	12
Assault	3
MVC	10
Blunt force	3
Pedestrian struck	1
Skateboard	2
Multi-trauma	16

Data presented as n (number)

Multi-trauma = TBI plus other non-CNS injuries

**Table 3:** Neuroradiologic features of DC patients

#	Age	CT description	Marshall score	Shift (mm)	ICP pre-DC	GOS	Contra. hydro	CT herniate	Cistern effaced	Craniectomy days post TBI	A-P Diameter bone flap
1	41	Bilat multi SA, SD, IP	6d	17	–	3	Trapped ventricle	U	Y	0	17
2	28	L multi, SA, SD, IP	6d	9	na	4	No	U	Y	6	18
3	47	L multi SD,IP, Cont	4	8	30	2	Temp horn	SF	Y	9	21
4	16	R temp lobe SD	4	6	–	4	Temp horn	SF	Y	0	21
5	45	L SA, SD, IP	3	4	–	4	Temp horn	No	Y	0	15
6	27	L multi IP, SD, SA	6d	7	25	1	No	SF	N	1	19
7	29	Bilat multi IP	6d	10	–	6	No	SF	Y	0	17
8	45	R multi SD, IP, active bleed	4	12	–	6	No	U	Y	0	15
9	53	L multi SD, cont, active bleed	6d	8	–	8	Temp horn	SF	Y	0	17
10	40	Bilat multi SA, SD, IP, active bleed	6d	15	–	7	No	TT	Y	0	14
11	56	R multi SA, SD, ED, IP	4	7	–	7	No	TT, U	Y	0	14
12	49	R multi SA, SD,sag. sinus. lac.	6d	7	–	1	No	SF	Y	0	14
13	21	Bilat multi IP, IV, active bleed	6d	0	34	8	No	SF	Y	2	19
14	46	Bilat multi SD, IP, SA, huge SD	6d	22	40	6	No	SF, U	Y	0	12
15	18	R front SA, SD, IP, hyperdense	6d	11	40	7	No	No	Y	4	16
16	43	Bilat IP, SD, SA	3	0	34	8	Trapprd ventricle	No	Y	12	16
17	39	Bilat SA, SD, IP, hyperdense	6d	5	30	8	No	No	Y	4	14
18	30	R occ., tem, SD, SA	6d	9	17	8	No	No	N	4	15
19	62	L mult IP, SD, mixed density	6d	22	–	6	Trapped ventricle	SF, U	Y	0	15
20	63	L multi IP, SD, SA	2c	3	–	7	No	No	Y	0	15
21	67	L multi SD, SA	6c	8	–	7	No	TT	N	1	15
22	57	L multi SD, IP, SA	4d	10	na	5	No	SF	N	1	16
23	25	Bilat multi SA, SD	4d	7	–	7	Yes	TT	Y	0	16
24	54	R multi SD, SA	2c	7	–	7	No	No	N	0	15
25	40	Bilat mult SD, SA, IP	6d	13	–	7	Trapped ventricle	SF	Y	0	14
26	72	L multi SD, SA, cont	2d	10	25	6	No	SF, U	N	3	15
27	19	L multi SD	4	8	–	3	No	No	N	0	13
28	28	Bilat multi ED, SA, IP	3	1	30	8	No	No	Y	0	15
29	23	Frontal cont.,SAH, SDH	2d	0	–	7	No	No	N	0	21
30	48	SDH + IPH	6d	9	30	8	No	No	Y	0	14
31	47	SDH, EDH, IPH	6d	4.9	42	7	No	SF	N	0	15

SA = subarachnoid, SD = subdural, IP = intraparenchymal, ED = epidural, cont. = contusion, sag. sinus lac. = sagittal sinus laceration, bilat multi. = bilateral multifocal, occ. = occipital, tem. = temporal, contra hydro = contralateral hydrocephalus, temp horn = temporal horn, front = frontal, U = uncal, SF = subfalcine, TT = transtentorial, na = not available

**Table 4:** Craniectomy A-P Diameter (cm)

A-P diameter (cm)	# Patients
11–12	4
13–14	7
15–16	10
17–18	7
>18	3

Mean A-P diameter 15.5 ± 2.9 cm

As expected with severe brain injury, early and late complications were common (Table 5). In the early post-DC period, central nervous system (CNS) events such as ICP elevations and seizures predominated. This was followed by electrolyte and respiratory issues including the adult respiratory distress syndrome (ARDS). Next to occur were infectious complications most notably pneumonia and urinary tract infections. CNS infections also occurred. Two patients developed mild hydrocephalus prior to cranioplasty. Cranioplasty was performed on 27 patients 2–12 weeks post-craniectomy (mean 7.2 ± 3.0 weeks). Cranioplasty was postponed in one patient for 15 weeks to allow completion of pregnancy and for 17 weeks in another due to severe intercurrent illness. Two patients died prior to cranioplasty. Following cranioplasty, one patient developed a surgical-site infection involving the bone-plate requiring its removal and subsequent prosthetic implant insertion. Another had increasing midline shift requiring bone-flap removal. A third patient sustained an acute subdural hemorrhage requiring evacuation. In the late follow-up period, 2 developed hydrocephalus requiring a VP shunt, 4 developed CSF leaks, and 4 developed late post-traumatic seizures). As a consequence of their injuries, 6 were left with a persistent partial hemiparesis; three others underwent drainage of chronic subdural hematomas.

**Table 5:** Complications Following DC

Early complications	n	Late complications	n
ICP elevation	8	Hydrocephalus	4
Ventriculitis/meningitis	3	Hemiparesis	7
Seizures	4	CSF leak	4
SDH requiring craniotomy	1	Post-traumatic seizures	11
Pneumonia/sinusitis	19	SDH requiring burr-hole	3
Other infection	13	Aphasia	6
Coagulopathy	3	Sympathetic storm	1
Electrolyte disorder	10	Post-ischemic optic neuritis	2
DVT/PE	3	Cranioplasty site infection	2
CHF/bradycardia	5	Cranioplasty requiring explant and prosthetic implant	1
Decubitus ulcer	3		
ARDS	7		
Dysautonomia	1		

The extended Glasgow outcome score (e-GOS) at 6 month’s post-injury was 7–8 (good to excellent) in 17 patients, 5–6 (moderate impairment) in 6 patients and poor outcome (Gouello et al. 2014; Brain Trauma Foundation 2007; Miller et al. 1977) in 6 patients (Table 6). Two patients died. Of the 6 survivors with poor outcomes (e-GOS = 2–4), five were the initial patients in the series. Given the small sample size, prognostic factors known to be associated with a favorable functional outcome such as age, time to craniectomy, ISS score, cerebral dominance, mm of midline shift, or size of the craniectomy flap could not be assessed accurately. There were no correlations (Pearson product-moment) between e-GOS and A-P diameter of the craniectomy, mm midline shift, or the modified Marshall grade. Only the initial GCS was significantly correlated by multiple logistic analyses. There were no differences in age, Marshall grade, mm midline shift, A-P diameter of the craniectomy flap or e-GOS in those patients in whom DC occurred more than 24 h following injury compared to patients undergoing immediate DC (Table 7).

**Table 6:** e-GOS

e-GOS	n
7–8	17
5–6	6
2–4	6
Died	2

**Table 7:** Clinical features of delayed DC patients

#	Age	CT description	Marshall score	Shift (mm)	ICP pre-DC	GOS	Contra. hydro	CT herniate	Cistern effaced	Craniectomy days post-TBI	A-P diameter bone-flap
2	28	L multi SA, SD, IP	6d	9	na	4	No	U	Y	6	17
3	47	L multi SD, IP, Cont	4	8	30	2	Temp Horn	SF	Y	9	21
6	27	L multi IP, SD, SA	6d	7	25	1	No	SF	N	1	19
13	21	Bilat multi IP, IV, active bleed	6d	0	34	8	No	SF	Y	2	19
15	18	R front SA, SD, IP, hyperdense	6d	11	40	7	No	No	Y	4	16
16	43	Bilat IP, SD, SA	3	0	34	8	Trapped ventricle	No	Y	12	16
17	39	Bilat SA, SD, IP, hyperdense	6d	5	30	8	No	No	Y	4	14
18	30	R occ, tem, SD, SA	6d	9	17	8	No	No	N	4	15
26	72	L multi SD, SA, cont	2d	10	25	6	No	SF, U	N	3	15

SA = subarachnoid, SD = subdural, IP = intraparenchymal, ED = epidural, cont. = contusion, sag. sinus lac. = sagittal sinus laceration, bilat multi. = bilateral multifocal, occ. = occipital, tem. = temporal, contra hydro = contralateral hydrocephalus, temp horn = temporal horn, front = frontal, U = uncal, SF = subfalcine, TT = transtentorial, na = not available

The review of recent DC studies reported during the past 10 years is tabulated in Table 8. Only the type of DC (FTP versus bi-frontal) predicted a good neurological outcome at 6-month [(odds ratio (O.R.) 0.43, 95 % confidence interval (CI) 0.31–0.60, p = 0.0001, z-statistic 4.74)]. Early DC (less than 24 h) compared to delayed DC (greater than 24 h) was not predictive. Complication rates were similar in the 10 references cited, as were the mortality rates, though the death rate in the present study was lower.

**Table 8:** Meta-analysis of Recent Literature Review (past 10 years) of DC for Severe TBI

Study author	Study year	# Patients	Study type	Mortality %	% e-GOS 6–8	FTP crani. %	Complication rate %	% time to DC < 24 h
Timofeev	2006	49	Retro	18	61	16	N/A	56
Aarabi	2006	50	Retro	28	40	98	84	98
Williams	2009	171	Retro	22	56	N/A	36	50
Qui	2009	37	Retro	27	57	100	30	100
Honeybul	2010	147	Retro	18	40	50	77	25
Chibbaro	2010	147	Prospe	23	67	88	23	100
Cooper	2011	73	Prospe	19	30	0	49	100
Nirula	2013	264	Retro	29	N/A	N/A	N/A	N/A
Gouello	2014	60	Retro	28	67	97	13	33
Present study		31	Retro	6	71	100	45	74
Totals	2006–2016	1029	8 retro 2 prospe	23	53	66	45	61

Association of FTP craniectomy with good to excellent outcome (e-GOS of 6–8). Odds Ratio (O.R.) = 0.43, 95 % confidence interval (CI) 0.31–0.60, p = 0.001, z-statistic 4.74

Retro = retrospective; Prospe = prospective; e-GOS 6–8 = good outcome; FTP crani = unilateral frontotemporoparietal craniectomy; DC = decompressive craniectomy

## Discussion

The malignant cerebral edema that attends severe TBI is a primary cause of poor neurological outcome and death. In addition to the mass effect of hematomas, the consequences of progressive cerebral swelling results in marked increases in intracranial pressure resulting in impaired cerebral perfusion (vascular effect) and brainstem herniation (mechanical effect) (Stocchetti and Maas 2014). As such, control of traumatic cerebral hypertension is the principle target of brain protective therapies (Brain Trauma Foundation 2007). In those TBI patients in whom ICP control can be achieved, neurological outcomes are generally better. In those who escape conventional treatment guidelines, alternate approaches are employed to prevent herniation and death. In the recently updated Brain Trauma Foundation Guidelines for the Management of Severe TBI, both hypothermia and decompressive craniectomy are second tier therapies to be considered. Hypothermia will lower ICP, but alone, has limited impact on adverse outcomes (Andrews et al. 2015). Unilateral DC, by allowing the swollen hemisphere to decompress through the craniectomy defect, lowers ICP, facilitating cerebral perfusion, and reduces the mechanical displacement of the brain (Gouello et al. 2014; Ecker et al. 2011; Aarabi et al. 2006).

Published results of DC studies have been variable owing in part to the heterogeneous nature of the reported studies. Earlier outcome reports of DC had unacceptably high morbidity and mortality rates (Nirula et al. 2014; Gouello et al. 2014). These studies, however, preceded the development of published guidelines for the management of severe TBI and frequent use of ICP monitoring. Adherence to these guidelines has lessened the burden of physiological responses known to strongly influence adverse outcomes such as hypoxia and hypotension (Chesnut et al. 1993; Chesnut 1997). Recent DC reports, albeit single center, retrospective, studies, have been more favorable. Most report good to excellent outcomes in 30–65 % of TBI patients subjected to this procedure (Gouello et al. 2014; Aarabi et al. 2006; Whitfield et al. 2001; Andrews et al. 2015; Guerra et al. 1999; De Luca et al. 2000; Gower et al. 1988; Qiu et al. 2009). Successful application also has been achieved in soldiers sustaining penetrating and blast injuries when subjected to bilateral or bi-compartmental DC (Ecker et al. 2011). Moreover, the efficacy of hemicraniectomy to increase survival without severe disability in young middle-cerebral-artery (MCA) stroke patients (DESTINY trial) adds further support to this procedure (Juttler et al. 2007). Although the 2009 Cochrane Collaboration literature review did not recommend DC in the adult trauma population for primary treatment because none of the included studies were randomized controlled trials (RCT), the authors did conclude that it could still be considered legitimate rescue therapy (Sahuquillo 2006). Improved GOS following DC was provided more recently in a randomized, population based study and by a prospective multicenter trial (Honeybul et al. 2010; Williams et al. 2009). In contrast, the 2011 NEJM RCT report of 155 patients, half of whom underwent bi-frontal craniectomy, showed no outcome benefit (in terms of e-GOS at 6-month) compared to medical therapy alone, as did a more recent propensity matched case–control trial of 264 patients who underwent DC (Nirula et al. 2014; Cooper et al. 2011). As a consequence of these results, enthusiasm for DC has fluctuated. The explanation for these contradictory results is multifactorial related, in part, to the heterogeneity of the population studied, the type of the study design, choice of surgical approach and operational factors. Yet, despite the successes and worldwide application of DC, the procedure remains controversial and there continue to be uncertainties regarding its appropriate application. The areas of controversy can be grouped into major categories including patient selection, timing of craniectomy, technical considerations, outcome results and complications.

Legitimate concerns have been raised in those studies reporting successful outcomes, including the present study, whether the procedure was performed on some who would have benefitted from a lesser procedure or medical management alone (Eghwrudjakpor and Allison 2010). Recent studies analyzing neurosurgical practice patterns and mortality for severe TBI in the state of Pennsylvania found that admission year between 2003 and 2013 to be significantly associated with DC (as preferential operative management for severe TBI compared to craniotomy), but with no improvement in the reported mortality (Morrison et al. 2016). Yet other studies have shown clear improvement in DC over craniotomy (De Luca et al. 2000; Morrison et al. 2016; Jiang et al. 2005; Kolias et al. 2016; Hartings et al. 2014; Li et al. 2012). In the present study, early DC (within 24 h post TBI) was performed as primary treatment when it was not possible to replace the bone flap or when specific clinical or neuroimaging criteria were met. These criteria included progression of hematoma size on early repeat CT imaging, rapidly rising ICP, progression of pupillary inequality, or persistent depression of P_bt_O_2_ unresponsive to medical manipulation (Joseph et al. 2014; Marini et al. 2014; Kim et al. 2014; Dawes et al. 2015).

Timing of DC remains a matter of controversy. Its purpose as a primary procedure is firstly the universally accepted indication of surgical evacuation of extra-axial hematoma, but also to relieve the pressure effect of brain contusion or edema, and to drain cerebrospinal fluid (Chibbaro et al. 2010). Clinical studies support the safety and effectiveness as a primary surgical procedure for these indications. Multiple recent studies including the present study report good outcomes and reduced mortality when DC is performed early following TBI (Gouello et al. 2014; Aarabi et al. 2006; Qiu et al. 2009; Chibbaro et al. 2010). The application of DC as a secondary procedure to control intracranial hypertension when medical management fails also has provoked dissent (Albanese et al. 2003). When used as rescue therapy, outcomes have been predictably worse. One of the elements driving this lack of consensus is the absence of clear guidelines regarding the indications and optimal timing of DC (Gouello et al. 2014; Albanese et al. 2003; Stein et al. 2013; Zhang et al. 2016; Reddy et al. 2002). Moreover, the application of multimodality monitoring including cerebral oxygenation (P_b_O_2_) and continuous EEG monitoring (c-EEG) and the influence of a dedicated NCC unit in which immediate responses including neurosurgical can be implemented promptly has not been consistently delineated in DC publications (Joseph et al. 2014; Marini et al. 2014; Kim et al. 2014; Dawes et al. 2015; Le Roux et al. 2014). More accurately, secondary DC is employed as a component of a tiered therapeutic protocol as a neuroprotective strategy rather than as a salvage procedure (Kolias et al. 2016).

Technical considerations of the unilateral DC have been extensively described (Huang and Wen 2010). It is essential that the craniectomy is of sufficient area to decompress the temporal lobe, but should be individualized on a patient-by-patient basis (Gower et al. 1988). It must not be too small such that brain tissue herniating through the defect can be further injured (Jiang et al. 2005). Evidence shows that sub-optimal bone windows (less than 12 cm) increases the chance of brain injury and poor outcome (Eghwrudjakpor and Allison 2010; Rossi-Mossuti et al. 2016; Liang et al. 2007; Wagner et al. 2001). In a recent Swiss study, there was considerable heterogeneity of the size of the bone flap (mean A-P diameter of 8.4 cm) with only 43 % of patients decompressed with a bone flap >12 cm. As a result, only 61 % of their patients had complete temporal decompression. It is possible that their 22 % mortality rate and 50 % complication rate was related to this technical featurel (Rossi-Mossuti et al. 2016). In the present study, in order to assure temporal decompression, the mean A-P diameter was 15.4 cm, and none were less than 12 cm (Table 3).

Choice of DC approach, bi-frontal or unilateral FTP has produced conflicting outcomes (Cooper et al. 2011; Whitfield et al. 2001; Polin et al. 1997). Although the selection of bi-frontal craniectomy may be dictated by the neuroimaging finding of diffuse edema without a predominant unilateral focus, often this is not the usual presentation of severe TBI. In the present study, all patients underwent unilateral DC. FTP DC allows for hemispheric decompression, evacuation of hematoma and severely contused brain and importantly, decompression of the temporal lobe base and cerebral cisterns. The meta-analysis of ten recent studies including the present study is consonant with recent literature showing improved outcomes and reduced mortality with unilateral DC (2,28,29,40, present study). In contrast, the randomized prospective study of bi-frontal DC reported no long term benefit outcomes compared to medical therapy alone (Cooper et al. 2011).

In the current study, 68 % of patients had a good to excellent outcome as measured by their e-GOS, and an overall mortality rate of 6 % (Table 5). The mortality reduction and better outcomes must be attributed to some degree, to the adequacy of decompression and to a neurosurgical staff experienced with this procedure. But, other factors also were operative including the strict observance to Guideline directed responses to elevated ICP, multimodality monitoring and to the multidisciplinary NCC unit with neurotrauma trained nursing staff who interceded rapidly whenever clinical conditions warranted (Brain Trauma Foundation 2007; Le Roux et al. 2014; Bouzat et al. 2015). Similar functional outcomes have been reported in contemporary series, though mortality rates in those successful studies were higher than in the present study and the e-GOS were assessed at a later time post-DC (36 months) (Gouello et al. 2014; Qiu et al. 2009; Williams et al. 2009; Chibbaro et al. 2010; Olivecrona et al. 2007). Physiologic improvement effecting the e-GOS may occur as much as 18 months’ post-injury whereas in the present study, the e-GOS was assessed only at 6 months’ post TBI. Furthermore, in the study reporting no incremental benefit of DC over craniotomy or ICP alone, the elements of neuromonitoring and their influence on clinical decision making were undocumented and may not have been uniformly utilized (Morrison et al. 2016).

As expected, multiple complications may follow unilateral craniectomy and cranioplasty (Table 4). The rates, though, are reported to be lower than with bi-frontal craniectomy (Gouello et al. 2014). These complications include subdural hygroma, dural sinus laceration, infection of the flap, hydrocephalus, post-traumatic seizures, CSF leaks, bone resorption of the flap necessitating additional reconstructive procedures or prosthetic replacement, and sinking skin flap syndrome causing paradoxical herniation. The complication rate and causes in this series were similar to those reported in earlier studies (Aarabi et al. 2006; Qiu et al. 2009; Chibbaro et al. 2010; Yang et al. 2008), but lower than other recent studies (Honeybul et al. 2013). Many complications including hemiparesis, post-traumatic seizures, dysautonomia, cognitive dysfunction and impulsivity also is related to the patient’s primary injury which DC will not reverse. Expectedly, these patients require intense medical care and careful surveillance not only for their cerebral injury, but also for the complications likely to occur with critical illness. Neurocognitive recovery is slow and prolonged neurological rehabilitation is expected. Complete behavioral, neurocognitive, and functional recovery rarely occurs following these devastating injuries and shouldn’t be anticipated. Acceptable functional recovery including return to work, school or activities preceding the TBI does occur and is the fundamental underpinnings of aggressive neurocritical care.

Craniectomy commits the patient to a minimum of one additional procedure (cranioplasty) which also can be a cause of complications. In the interim, a helmet or other protective gear must be worn to avoid further brain injury from inadvertent falls or other traumas (Gouello et al. 2014; Aarabi et al. 2006; Vespa et al. 1999). If considerable depression of the skin at the site of the cranial defect occurs coupled with progressive neurological deterioration which improves with recumbency, strong consideration should be given to immediate replacement of the bone flap provided the patient is otherwise stable and the scalp is free of infection (Yang et al. 2008; Sarov et al. 2010).

Cranioplasty was conducted when the patient was clinically improved, but would be best done as early as possible since atmospheric pressure may cause local vascular dysfunction (Chibbaro et al. 2010). One prospective multicenter trial demonstrated that cranioplasty improved brain perfusion which may have led to improved neurocognitive function and, therefore, should be performed early following DC (Chibbaro et al. 2013). Most DC studies recommend a time-frame of 12 weeks. In the present study, as in the prospective multicenter study cranioplasty was performed at 7.7 ± 3.7 weeks following injury (Chibbaro et al. 2013).

In the present study of unilateral DC, only an initial GCS > 5 was determinant of a good long-term neurological outcome by logistic regression analysis (Gouello et al. 2014). The meta-analysis of ten recent DC studies also confirms that the FTP DC approach is preferable when this is technically possible (Nirula et al. 2014; Gouello et al. 2014; Cooper et al. 2011; Aarabi et al. 2006; Qiu et al. 2009; Honeybul et al. 2010; Williams et al. 2009; Chibbaro et al. 2010; Timofeev et al. 2006 present study). Meta-analyses must be interpreted with caution due to selection bias in the included studies, heterogeneity of methodology and study design. Many of the included studies reported outcomes from both bi-frontal and unilateral approaches and early and delayed DC. It was not always possible to assign a specific outcome, mortality or e-GOS, based on timing of DC or operative technique. Given the small sample size, factors known to be associated with neurological outcome following DC including age, time to craniectomy, concomitant non-neurological trauma (higher ISS scores), Marshall score, mm of midline shift, and dimensions of the craniectomy were not found to be predictive of mortality, complication rate or e-GOS as others have reported (Gouello et al. 2014; Aarabi et al. 2006; Rossi-Mossuti et al. 2016). Dominant hemisphere involvement did negatively impact on good outcome, but didn’t quite reach statistical significance (p < 0.08; OR 0.003). DC performed on the day of injury in 22 study patients was dictated by the intra-operative findings of extra-axial hematoma, brain herniation and edema. Subsequent DC in the other 9 patients was dictated by failure of medical management to control intracranial hypertension or clinical progression. There were no differences in the clinical or radiologic characteristics of those undergoing immediate DC and those in whom DC was delayed. Unfavorable outcome and mortality was not different between the two groups, though the small sample size does not permit valid statistical inferences. One meta-analysis of 5 studies comparing early and delayed DC also found no statistical differences (Zhang et al. 2016). In that report bilateral non-reactive pupils correlated with poor outcome and death. In the present study we excluded those patients from consideration for DC.

This study was undertaken, in part, as a validation of prior successful DC studies (given the recent negative studies). Our functional outcomes as assessed by the e-GOS were better than most previously reported series and the mortality rate was low. Whether it should be applied as initial therapy or only for rescue of refractory ICP is unsettled. In a recently reported epidemiologic study of 22,229 TBI patients of which 5406 were designated as critically head injured and underwent operative treatment between 2003 and 2013, a significant increase in adjusted mortality was found over the time period of the study despite adaptations in neurosurgical practice toward more DC and less craniotomies (Morrison et al. 2016). Unfortunately, this study did not delineate the extent of the craniectomy, whether temporal decompression was accomplished, or the application and influence of neuromonitoring on clinical decision-making. In the Swiss study referenced above, only 61 % had adequate decompression of the basilar cisterns (Rossi-Mossuti et al. 2016). We believe that the technique of DC that assures temporal decompression is essential to improving the outcome of these severely injured patients. This technique coupled with guideline-driven care in a multidisciplinary NCC unit with the capability of multi-modality monitoring was likely responsible for our favorable results.

The major limitation of this study is that it is retrospective and observational and the sample size is small. No randomization or blinding of study participants was intended (or possible). The authors are aware of the prospective, large multicenter trial of DC (RESCUEicp) (Hutchinson et al. 2011). Additionally, given the small sample size, we could not distinguish patients who underwent primary DC (within 24 h of injury) from those who underwent DC as rescue therapy (after 24 h), as a means of controlling ICP.

## Conclusion

This study describes 31 patients who underwent unilateral DC for TBI. In 22 of these patients DC was the initial surgical intervention while in the other 9 patients DC was performed after failure of medical management to control cerebral hypertension. 22 of 31 patients (71 %) had a good to moderate outcome, 6 of 31 (19 %) had a poor neurological outcome and 2 patients (6 %) died. These results are highly encouraging and compare favorably to other published reports of DC, but with lower mortality (Gouello et al. 2014; Aarabi et al. 2006; Whitfield et al. 2001). Thus, in this series of severe but survivable TBI in adults, a good to excellent result was obtained in most patients following decompressive craniectomy with an acceptable morbidity and mortality.

## References

[CR1] Aarabi B, Hesdorffer DC, Ahn ES, Aresco C, Scalea TM, Eisenberg HM (2006). Outcome following decompressive craniectomy for malignant swelling due to severe head injury. J Neursurg.

[CR2] Albanese J, Leon M, Alliez JR (2003). Decompressive craniectomy for severe traumatic brain injury: evaluation of the effects at one year. Crit Care Med.

[CR3] Andrews PJD, Sinclair HL, Rodriguez A, Harris BA, Battison CG, Rhodes JKL, Murray GD (2015). Hypothermia for intracranial hypertension after traumatic brain injury. NEJM.

[CR4] Bouzat P, Marquez JB, Sala N (2015). Accuracy of brain multimodal monitoring to detect cerebral hypoperfusion after traumatic brain injury. Crit Care Med.

[CR5] Brain Trauma Foundation (2007). Guidelines for the management of severe traumatic brain injury. J Neurotrauma.

[CR6] Chesnut RM (1997). The management of severe traumatic brain injury. Emerg Med Clin North Am.

[CR7] Chesnut RM, Marshall LF, Klauber MR, Blunt BA, Baldwin N, Eisenberg HM (1993). The role of secondary brain injury in determining outcome from severe head injury. J Trauma.

[CR8] Chibbaro S, Di Rocco F, Mirone G, Fricia M (2010). Decompressive craniectomy and early cranioplasty for the management of severe head injury: a prospective multicenter study on 147 patients. World Neurosurg.

[CR9] Chibbaro S, Vallee F, Beccaria K, Poczos P (2013). The impact of early cranioplasty on cerebral blood flow and its correlation with neurological and cognitive outcome. Prospective multicenter study on 24 patients. Rev Neuro (Paris).

[CR10] Cooper DJ, Rosenfeld JV, Murray L, Arabi YM, Davies AR, D’Urso P, Kossmann T, Ponsford J, Seppelt I, Reilly P, Wolfe R (2011). Decompressive craniectomy in diffuse traumatic brain injury. NEJM.

[CR11] Dawes AJ, Sacks GD, Cryer G, Ko C, for the Los Angeles County Trauma Consortium (2015). Intracranial pressure monitopring and inpatient mortality in severe traumatic brain injury: a propensity score-matched analysis. J Trauma.

[CR12] De Luca GP, Volpin L, Fornezza U, Cervellini P, Zanusso M, Casentini L (2000). The role of decompressive craniectomy in the treatment of uncontrollable post-traumatic intracranial hypertension. Acta Neurochir Suppl.

[CR13] Ecker RD, Mulligan LP, Dirks M, Bell RS, Severson MA, Howard RS, Armonda RA (2011). Outcomes of 33 patients from the wars in iraq and Afganistan undergoing bilateral or bicompartmental craniectomy. J Neurosurg.

[CR14] Eghwrudjakpor PO, Allison AB (2010). Decomprssive craniectomy following brain injury: factors important to patient outcome. Libyan J Med.

[CR15] Gouello G, Hamel O, Aschnoune K, Bord E, Robert R, Buffenoir K (2014). Study of the long-term results of decompressive craniectoomy after severe traumatic brain injury base on a series of 60 consecutive cases. Sci World J.

[CR16] Gower DJ, Lee KS, McWhorter JM (1988). Role of subtemporal decompression in severe closed head injury. Neurosurgery.

[CR17] Guerra WK, Gaab MR, Dietz H, Mueller JU, Piek J, Fritsch MJ (1999). Surgical decompression for traumatic brain swelling: indications and results. J Neurosurg.

[CR18] Hartings JA, Vidgeon S, Strong AJ (2014). Surgical management of traumatic brain injury,: a comparative-effectiveness study of 2 centers. J Neurosurg.

[CR19] Honeybul S, Ho KM, Lind CR, Gillett GR (2010). Observed versus predicted outcome for decompressive craniectomy: a population based study. J Neurotrauma.

[CR20] Honeybul S, Janzen C, Kruger K, Ho KM (2013). Decompressive craniectomy for severe traumatic brain injury: is life worth living. J Neurosurg.

[CR21] Huang X, Wen L (2010). Technical considerations in decompressive craniectomy in the treatment of traumatic brain injury. Int J Med Sci.

[CR22] Hutchinson PJ, Kolias AG, Timofeev I, Corteen E (2011). Update on the RESCUEicp trial. Crit Care.

[CR23] Jiang JY, Xu W, Li WP (2005). Efficacy of standard trauma craniectomy for refractory intracranial hypertension with severe traumatic brain injury: a multicenter, prospective, randomized controlled study. J Neurotrauma.

[CR24] Joseph B, Friese RS, Sadoun M, Rhee P (2014). The BIG (brain injury guidelines) project: defining the management of traumatic brain injury by acute care surgeons. J Trauma.

[CR25] Juttler E, Schwab S, Schmiedek P (2007). Decompressive surgery for the treatment of malignant infarction of the middle cerebral artery (DESTINY) a randomized, controlled trial. Stroke.

[CR26] Juttler E, Unterberg A, Woitzik J, Bosel J, Amiri H, Sakowitz OW, Gondan M, Schiller P, Limprecht R, Luntz S, Schneider H, Pinzer T, Hobohm C, Meixensberger J, Hacke W (2014). Hemicraniectomy in older patients with extensive middle-cerebral-artery stroke (DESTINY II). NEJM.

[CR27] Kim DR, Yang SH, Sung JH, Lee SW (2014). Significance of intracranial pressure monitoring after early decompressive craniectomy in patients with severe traumatic brain injury. J Korean Neurosurg Soc.

[CR28] Kjellberg RN, Prieto A (1971). Bifrontal decompressive craniotomy for massive cerebral edema. J Neurosurg.

[CR29] Kocher T (1901) Die therapies des hirndrucks. In: Hirnerschuetterung, Hirdruck und Chirurgische EingriffebBei Hirnkrankheiten. A. Hoelder, Steyr, pp 262–266

[CR30] Kolias AG, Adams H, Timofeev I, Czosnyka M (2016). Decompressive craniectomy following traumatic brain injury: developing the evidence base. Br J Neurosurg.

[CR31] Le Roux P, Menon DK, Citerio G, Vespa P, Bader MK (2014). Consensus summary statement of the international multidisciplinary consensus conference on multimodality monitoring in neurocritical care. Neurocrit Care.

[CR32] Li JM, Kolias AG, Guilfoyle MR (2012). Outcome following evacuation of subdural hematomas: a comparison of craniotomy with decompressive craniectomy. Acta Neurochir (Wien).

[CR33] Liang W, Xiaofeng Y, Weiguo L (2007). Cranioplasty of large cranial defect at an early stage after decompressive craniectomy performed for severe head trauma. J Craniofac Surg.

[CR34] Marini CP, Stoller C, Shah O, Policastro A (2014). The impact of early flow and brain oxygen crisis on the outcome of patients with severe traumatic brain injury. Am J Surg.

[CR35] Miller JD, Becker DP, Ward JD, Sullivan HG, Adams WE, Rosner MJ (1977). Significance of intracranial hypertension in severe head injury. J Neurosurg.

[CR36] Mitchell P, Tseng M, Mendelow AD (2004). Decompressive craniectomy with lattice duroplasty. Wien.

[CR37] Morrison CA, Gross BW, Cook AD, Estrella L (2016). An analysis of neurosurgical practice patterns and outcomes for serious to critical traumatic brain injuries in a mature trauma state. J Trauma Acute Care Surgery.

[CR38] Nirula R, Millar D, Greene T, McFadden M, Shah L, Scalea TM, Stein DM, Magnotti LJ, Jurkovich GJ, Vercruysse G, Demetriades D, Scherer LA, Peitzman A, Sperry J, Beauchamp K, Bell S, Feiz-Erfan I, O’Neill P, Coimbra R (2014). Decompressive craniectomy or medical management for refractory intracranial hypertension: an AAST-MIT propensity score analysis. J Trauma ACS.

[CR39] Olivecrona M, Rodling-Wahlstrom M, Naredi S, Koskinen LO (2007). Effective ICP reduction by decompressive craniectomy in patients with severe traumatic brain injury treated by an IVP-targeted therapy. J Neurosurg.

[CR40] Polin RS, Shaffrey ME, Bogaev CA (1997). Decompressive bifrontal craniectomy in the treatment of severe refractory posttraumatic cerebral edema. Neurosurgery.

[CR41] Qiu W, Guo C, Shen H, Chen K, Wen L, Huang H, Ding M, Sun L, Jiang Q, Wang W (2009). Effects of unilateral decompressive craniectomy on patients with unilateral acute post-traumatic brain swelling after severe traumatic brain injury. Crit Care.

[CR42] Ransohoff J, Benjamin MV, Gage EL, Epstein F (1971). Hemicraniectomy in the management of acute subdural hematoma. J Neurosurg.

[CR43] Reddy AK, Saradhi V, Panigrahi M, Rao TN (2002). Decompressive craniectomy for stroke: indications and results. Neurol India.

[CR44] Rossi-Mossuti F, Fisch U, Schoettker P, Gugliotta M (2016). Surgical treatment of severe traumatic brain injury in Switzerland: results from a multicenter study. J Neurolog Surg.

[CR45] Sahuquillo J (2006). Decompressive craniectomy in the treatment of refractory high intracranial pressure in traumatic brain injury (review). Cochran Coll.

[CR46] Sarov M, Guichard JP, Chibarro S, Guittard E, Godin O, Yelnik A, George B (2010). Sinking skin flap syndrome and paradoxical herniation after hemicraniectomy for malignant hemispheric infarction. Stroke.

[CR47] Stein DM, Brenner M, Hu POF, Scalea TM (2013). Timing of intracraninal hypertension following severe traumatic brain injury. Neurocrit Care.

[CR48] Stocchetti N, Maas AIR (2014). Traumatic intracranial hypertension. NEJM.

[CR49] Timofeev I, Kirkpatrick PJ, Corteen E, Hiler M (2006). Decompressive craniectomy in traumatic brain injury: outcome following protocol-driven therapy. Acta Neurochir.

[CR50] Venes JL, Collins WF (1975). Bifrontal decompressive craniectomy in the management of head trauma. J Neursurg.

[CR51] Vespa P, Nuwer MR, Nenov V (1999). Increased incidence and impact of non-convulsive and convulsive seizures after traumatic brain injury as detected by continuous electroencephalographic monitoring. J Neurosurg.

[CR52] Wagner S, Schnippering H, Aschoff A (2001). Suboptimal craniectomy as a cause of additional cerebral lesions in patients with malignant infarct of the MCA. J Neurosurg.

[CR53] Whitfield PC, Patel H, Hutchinson JA, Czosnyka M, Parry D, Menon D, Pickard JD, Kirkpartick PJ (2001). Bifrontal decompressive cranietcomy in the management of posttraumatic intracranial hypertension. Br J Neurosurg.

[CR54] Williams RF, Magnotti IJ, Croce MA, Hargraves BB, Fisher PE (2009). Impact of decompressive craniectomy on functional outcome after severe traumatic brain injury. J Trauma.

[CR55] Yang XJ, Hong GL, Su SB (2003). Complications induced by decompressive craniectomies after traumatic brain injury. Chin J Traumatol.

[CR56] Yang XF, Wen L, Shen F (2008). Surgical complications secondary to decompressive craniectomy in patients with a head injury: a series of 108 consecutive cases. Acta Neurochir.

[CR57] Zhang K, Jiang W, Ma H (2016). Comparison of early and late decompressive craniectomy on the long-term outcome in patients with moderate and severe traumatic brain injury: a meta-analysis. Br J Neurosurg.

